# FTIR Metabolomic Fingerprint Reveals Different Modes of Action Exerted by Structural Variants of *N*-Alkyltropinium Bromide Surfactants on *Escherichia coli* and *Listeria innocua* Cells

**DOI:** 10.1371/journal.pone.0115275

**Published:** 2015-01-14

**Authors:** Laura Corte, Matteo Tiecco, Luca Roscini, Sergio De Vincenzi, Claudia Colabella, Raimondo Germani, Carlo Tascini, Gianluigi Cardinali

**Affiliations:** 1 Department of Pharmaceutical Sciences—Microbiology, University of Perugia, Borgo XX Giugno 74, I-06121 Perugia, Italy; 2 CEMIN, Centre of Excellence on Nanostructured Innovative Materials, Department of Chemistry, Biology and Biotechnology, University of Perugia, via Elce di Sotto 8, I-06123 Perugia, Italy; 3 U.O. Malattie Infettive, Azienda Ospedaliera Universitaria Pisana, Via Paradisa 2, Cisanello, 56100 Pisa, Italy; Korea University, REPUBLIC OF KOREA

## Abstract

Surfactants are extremely important agents to clean and sanitize various environments. Their biocidal activity is a key factor determined by the interactions between amphiphile structure and the target microbial cells. The object of this study was to analyze the interactions between four structural variants of *N*-alkyltropinium bromide surfactants with the Gram negative *Escherichia coli* and the Gram positive *Listeria innocua* bacteria. Microbiological and conductometric methods with a previously described FTIR bioassay were used to assess the metabolomic damage exerted by these compounds. All surfactants tested showed more biocidal activity in *L. innocua* than in *E. coli*. *N*-tetradecyltropinium bromide was the most effective compound against both species, while all the other variants had a reduced efficacy as biocides, mainly against *E. coli* cells. In general, the most prominent metabolomic response was observed for the constituents of the cell envelope in the fatty acids (W1) and amides (W2) regions and at the wavenumbers referred to peptidoglycan (W2 and W3 regions). This response was particularly strong and negative in *L. innocua*, when cells were challenged by *N*-tetradecyltropinium bromide, and by the variant with a smaller head and a 12C tail (*N*-dodecylquinuclidinium bromide). Tail length was critical for microbial inhibition especially when acting against *E. coli*, maybe due the complex nature of Gram negative cell envelope. Statistical analysis allowed us to correlate the induced mortality with the metabolomic cell response, highlighting two different modes of action. In general, gaining insights in the interactions between fine structural properties of surfactants and the microbial diversity can allow tailoring these compounds for the various operative conditions.

## Introduction

Surfactants are among the worldly most common chemicals, widely used as detergents [1], paints [2, 3], emulsificants [4], de-mulsificants [5] and metal flotation agents [6]. Their structural characteristics allow the dissolution in both polar and apolar solvents thanks to the presence of hydrophilic head groups (cationic, anionic, zwitterionic or non-ionic) and hydrophobic moiety [7]. Surfactants can be synthesized chemically [8, 9, 10, 11, 12] or can be obtained from bacteria and fungi [13].

The use of surfactants as biocides against bacteria, virus and fungi is one of their most appreciated features [8, 14, 15, 16, 17, 18, 19]; in fact many such molecules are used as antibiotics against Gram negative and Gram positive multi drug-resistant bacteria, e.g. insensitive to colistin and daptomycin [20].

Many studies have been dedicated to elucidate the mechanisms governing this biocidal activity. In spite of these efforts, there is not yet a full consensus on their mode of action. According to a long lasting model, the primary targets of cationic surfactants are supposedly the phospholipids components of the bacterial cell membrane, somehow destabilized by surfactants, leading to membrane distortion followed by decompartimentalization and cell lysis [21, 22, 23]. Additionally, the negative charge on the bacterial cells seems to be involved in this process through an ion exchange mechanism by the cytoplasmic membrane and the cationic surfactants [24]. It was also suggested that the length, rather than the composition of the alkyl chain, plays a primary role in surfactants biocidal activity [25, 26]. The antimicrobial surfactants effect depends from their hydrophilic/lipophilic balance, but the efficacy appears to be modulated by the composition of the cell envelope, and particularly of the cell membrane, in a strain-dependent manner [8]. Additionally, endo-metabolites (e.g. superoxides) can be produced in response to surfactants [27]. Altogether, it seems that the mode of action is the complex result of surfactant structure and cell type used as target.

Fourier Transform InfraRed Spectroscopy (FTIR) has been applied in microbiological studies to whole cell analysis [12, 28, 29, 30, 31]. A FTIR-based bioassay was developed in our laboratory to determine the presence and the extent of cellular stress, with the rationale that stressing conditions can alter the cell metabolome before and after cell death [32]. This assay was tested primarily against yeast cells, to detect the types of molecules more involved in the stress phenomenon [33], but was also extended to mammal cells [34] similarly to other approaches aiming at determining environmental stress [35, 36]. These features make FTIR metabolomics fingerprint a tool of choice to characterize the biological activity exerted by surfactants.

A practical approach to the study of surfactants antimicrobial properties is to consider that there are many contaminants in environments and situations calling for simultaneous cleaning and effective microbial inactivation. These two activities can be exerted by surfactants, provided that their structure combines effectively detergency and biocidal effect against the typical spoilers and contaminants of the environment to sanitize. *E. coli* and *L. innocua* represent dangerous bacterial contaminants [37, 38, 39] and a model to study biological effects against Gram negative and Gram positive bacteria, respectively.

A series of structural variants of *N*-alkyltropinium bromide surfactants has been previously synthesized and tested against not pathogenic or opportunistic yeasts, demonstrating that alkyl tail length and head-group size affected their anti-microbial activity [40]. Four of these structural variants were employed in the present study to test whether FTIR spectroscopy can differentiate their modes of action at the metabolomic level and evaluate their efficacy against a Gram + and Gram—bacterial species. Cell damage was evaluated after 1 hr exposition in order to exclude any problem deriving from the limitations due to compounds diffusion in the agar medium and to exclude the mortality and physiological changes deriving from a long lasting deprivation of nutrients [41]. Statistical analysis allowed to propose a typing of the mode of action based on the biocidal activity and on the metabolomic damage exerted by stressing compounds. The possibility to generalize the typing obtained in this study will require more insight with compounds other than those considered in this paper. This is potentially important for high-throughput first level screening of novel putative biocidal compounds. An analysis of the functional FTIR data significance showed that it is possible to dissect a *ante mortem* from a *post mortem* cell metabolomic damage.

## Materials and Methods

### Bacterial strains and growth conditions

Bacterial strains *E. coli* BCF 2 and *L. innocua* BCF 13 were obtained from the internal collection of the Microbial Genetics and Phylogenetics Laboratory of the Department of Pharmaceutical Sciences (University of Perugia). They are deposited in the Netherlands Culture Collection of Bacteria (NCCB) as NCCB 100509 and NCCB 100510 respectively.

Pre-cultures and subsequent cultures were inoculated at OD_600_ = 0.2 in 100 mL of BHI medium (Brain Heart Infusion—Biolife Italiana S.r.l., Milan, Italy) and grown 24 h at 37°C, with 150 rpm shaking, leading the cultures to cell densities around 4*10^8^ cells/mL. The biomass amount for the FTIR analysis was normalized using the optical densities at 600 nm, taking care to read the cell suspensions in the OD_600_ = 0.1 to 0.5 to guarantee the linearity between the cells and the optical density. The actual optical density was calculated by multiplying the dilution factor by the recorded OD_600_.

### Synthesis and purification of quaternary ammonium salts

The tropinium-head surfactants were synthesized by quaternization of the tropine with the corresponding n-alkyl bromide heated to reflux in acetonitrile. The *23S*-sh surfactant was synthesized from quinuclidine and n-dodecyl bromide in acetonitrile at room temperature (Table 1 and S1 Table). All detailed synthetic procedures and characterizations were reported in previous papers [40, 42].

**Table 1:** Structures, *c.m.c.* and α values of quaternary ammonium salts.

		**M.W.**	***c.m.c.* (M)**	**α**
***23S*-12**	*N*-dodecyltropinium bromide	390.4	1.14 · 10^-2^	0.30
***23S***	*N*-tetradecyltropinium bromide	418.5	2.62 · 10^-3^	0.28
***23S*-16**	*N*-hexadecyltropinium bromide	446.5	[n.a. due to low solubility]	[n.a. due to low solubility]
***23S*-sh**	*N*-dodecylquinuclidinium bromide	360.42	1.08·10^-2^	0.25

### FTIR-based bioassay

Each cells suspension was centrifuged (3 min at 5300 *g*), washed twice with distilled sterile water and re-suspended in six polypropylene tubes with an appropriate amount of distilled water (standardized optical density OD_600_ = 12). Surfactants were added to the test tubes in order to obtain the concentrations of 0.2, 0.4, 0.6, 0.8 and 1.0 mM. A maximum 1.0 mM concentration was chosen on the basis of the outcomes of a previous work [42]. The control (0.0 mM) was obtained by re-suspending the cells directly in distilled sterile water. All tests were carried out in triplicate. Tubes were incubated 1 hr at 25°C in a shaking incubator set at 50 rpm [41]. After the incubation, 1.5 mL suspension was taken from each sample, centrifuged (5 min at 5,300 *g*) washed twice with distilled sterile water and re-suspended in 1.5 mL HPLC grade water. 105 µL suspension was sampled for three independent FTIR readings (35 µL each, according to the technique suggested by Manfait and co-workers [43]). The FTIR experiments were carried out with a TENSOR 27 FTIR spectrometer, equipped with HTS-XT accessory for rapid automation of the analysis (BRUKER Optics GmbH, Ettlingen, Germany). FTIR measurements were performed in transmission mode. All spectra were recorded in the range between 4000 and 400 cm^-1^. Spectral resolution was set at 4 cm^-1^, sampling 256 scans per sample. The software OPUS version 6.5 (BRUKER Optics GmbH, Ettlingen, Germany) was used to carry out the quality test, baseline correction, vector normalization and the calculation of the first and second derivatives of spectral values.

### Spectra statistical analyses

The MSA (Metabolomic Spectral Analysis) script employed in this study was developed in “R” language to carry out the following operations on the matrices of spectral data exported as ASCII text from OPUS 6.5. The analytical process consists in calculating the distance between the spectrum of the cells under test and that of the cells without the stressing agent; this procedure is extended to the five spectral regions in order to differentiate the stress response among the different classes of molecules [32].

In more detail, the procedure could be outlined as follows:

1. Each single spectrum was normalized in the range spanning from 0 to 1, in a way already suggested by Goodacre and coll. [44]. Average spectra from the three repetitions were calculated.
2. Response spectra (hereinafter reported as RS) were calculated as difference between each average spectrum and the average spectrum of the same cells maintained in water (defined as control RS). Response spectra of each agent were plotted with the exclusion of the control RS, which is by definition a straight line with RS = 0.
3. Synthetic stress indexes (hereinafter reported as SI) of metabolomic stress response were calculated as Euclidean distances of the RS under stress and the control RS. SI of the whole spectrum and of the five different spectral regions individuated by Kuemmerle et al. [45] were calculated. The five regions were defined as follows: fatty acids (W1) from 3000 to 2800 cm^-1^, amides (W2) from 1800 to 1500 cm^-1^, mixed region (W3) from 1500 to 1200 cm^-1^, carbohydrates (W4) from 1200 to 900 cm^-1^ and typing region (W5) from 900 to 700 cm^-1^. Since the five spectral regions differ in length, their SI were scaled to the length of the whole spectrum, in order to make the different SI comparable on the same scale.

### Biocidal activity test

The biocidal activity tests were carried out in parallel with the FTIR based stress bioassay to compare the metabolomic damages with the loss of viability. 100 µL of each cells suspension prepared for the FTIR analysis were serial diluted to determine the viable cell counting, in triplicate, on BHI plates. The biocidal effect of the tested compounds was highlighted as cell mortality induced at different concentrations. The cell mortality (M) was calculated as M = (1-Cv/Ct) x 100, where Cv is the number of viable cells in the tested sample and Ct the number of viable cells in the control suspension.

### Conductivity measurements of *N*-tetradecyltropinium bromide solution with bacterial cells

Conductivity was measured on a CRISON GLP-31 conductivity meter at 25.0 ± 0.1°C (Pharmacia Biotech Multitemp III thermostat). A Watson-Marlow 323 peristaltic pump (with Watson-Marlow tubes) was used for the surfactant solutions and cell suspensions additions. A JASCO V-530 spectrophotometer was used to determine cell suspension concentration. Cell suspensions were prepared by washing cell pellets twice with HCl 5·10^-3^ M and three times with bi-distilled water to standardize the supernatant conductivity to a value below 10 μS/cm. Each cell suspension was prepared at a standardized optical density OD_600_ = 5. *N*-tetradecyltropinium bromide stock solution was prepared at 0.02 M. Conductivity measurements were carried out using the automated method proposed by Tiecco and co-workers [46]. Data were imported in R statistical environment (http://cran.r-project.org/) and treated with the CMC-R script, freely available from the following sites:http://www.bio-aware.com/BioloMICSNews.aspx?Rec=87 and http://docs.google.com/file/d/0Bzj3M187nEU2YlRKbVB5cnBtWEk/edit?usp=sharing.

## Results

### Metabolomic characterization of the stress induced by *N*-alkyltropinium bromide structural variants

**Figure 1:**
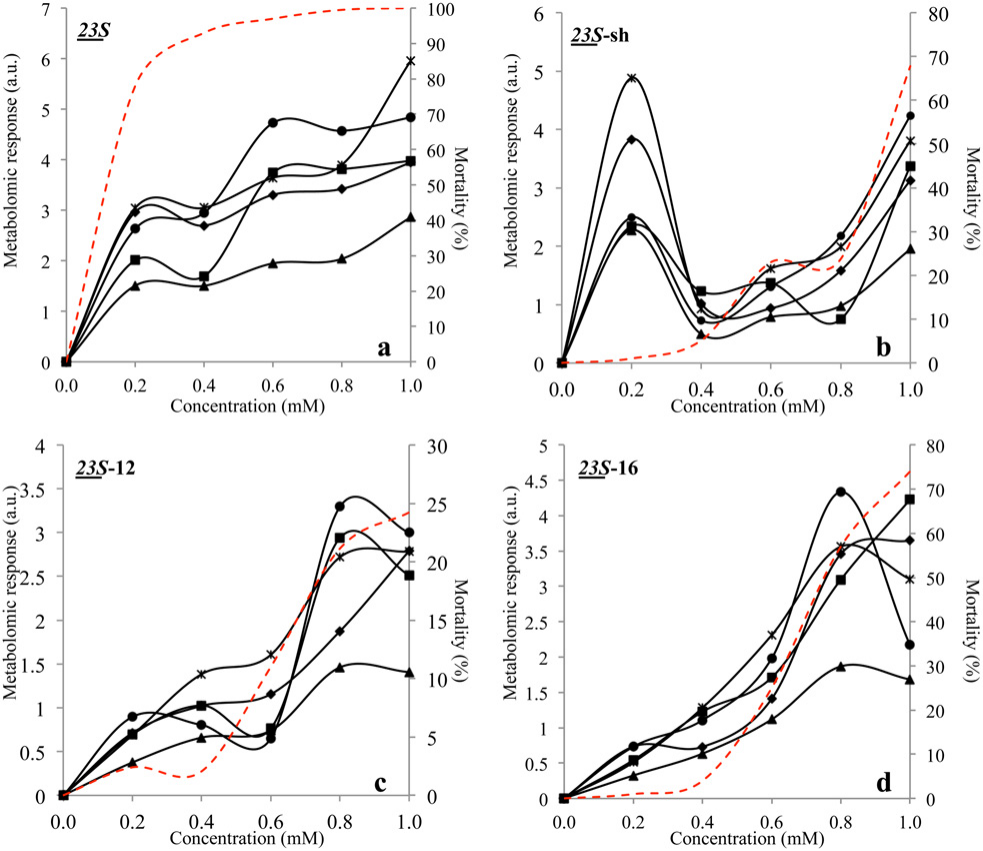
Stress Indexes of *E. coli* cells subjected to the action of *N*-alkyltropinium bromide structural variants at 0.2, 0.4, 0.6, 0.8 and 1.0 mM. a.u. stands for *“arbitrary units”*; triangles represent the whole spectrum, asterisks W1 region, diamonds W2 region, squares W3 region, circles W4 region; dashed line represents mortality. The degree of variability between replicas throughout the FTIR spectra ranged around 2.3*10^-2^.

**Figure 2:**
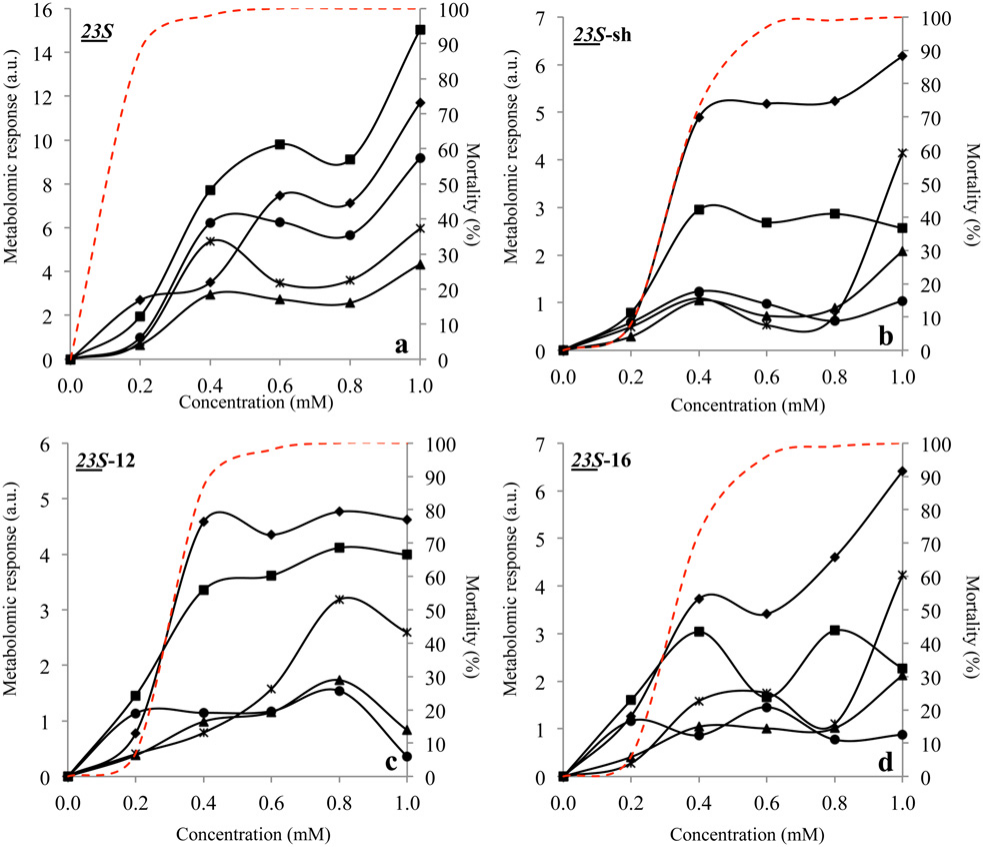
Stress Indexes of *L. innocua* cells subjected to the action of *N*-alkyltropinium bromide structural variants at 0.2, 0.4, 0.6, 0.8 and 1.0 mM. a.u. stands for *“arbitrary units”*; triangles represent the whole spectrum, asterisks W1 region, diamonds W2 region, squares W3 region, circles W4 region; dashed line represents mortality. The degree of variability between replicas throughout the FTIR spectra ranged around 2.7*10^-2^.

The analysis of the stress induced by the *23S* surfactant and the other variants on *E. coli* and *L. innocua* cells was carried out by calculating Stress Indexes previously defined as the variation of specific spectral areas caused by the exposition of cells to each surfactant [32]. For this analysis, only four regions were considered out of the five analyzed with MSA. The typing region (900–700 cm^-1^) was omitted because its response did not appear correlated with these specific stressing conditions. *23S* proved the most effective against both bacterial species, with a greater biocidal efficacy in *L. innocua* in respect to *E. coli* (Figs. 1a and 2a). In fact, it caused 100% mortality to *L. innocua* already at 0.2 mM, with SI ranging from 0 to 15, representing approximately the double of the corresponding figures obtained with *E. coli*. All SIs increased from 0.1 mM with similar trends. Interestingly, the steepest increase was observed from 0.0 and 0.2 mM, although the SIs from W3 and W4 showed a secondary steep increase between 0.4 and 0.6 mM and W1 from 0.8 and 1.0 mM. The steep increase from 0.0 to 0.2 mM corresponds to a similar raise of the mortality from 0 to ca. 80%, suggesting that all regions registered the stress causing the mortality of most of the cells (Fig. 1a).

All the SIs curves showed an increasing trend from 0.0 to 0.4 mM, a concentration causing 100% mortality. As for *E. coli,* all regions registered the stress leading the cells to die. Interestingly, there is another increase of all SIs from 0.8 to 1.0 mM clearly due to *post mortem* effects (Fig. 2a). In both species the Global Stress Index referred to the whole spectrum resulted GSI > 1.0 at every concentration of the surfactant, confirming that this index is a reliable marker of effective cell stress when reaching values above 1.0 [30, 32].

*23S-*sh induced low mortality rates in *E coli* ranging from 0 at 0.2 mM to less than 70% at 1 mM, presenting a local peak of 25% mortality at 0.6 mM (Fig. 1b). Interestingly, all SIs add a maximum at 0.2 mM, decreased at 0.5 mM and increased slowly at 1.0 mM, reaching values similar or lower than those observed at 0.2 mM. This phenomenon can be explained by the fact that, at low surfactant concentration, cells responded actively to the presence of the surfactant without suffering significant mortality. Interestingly, the most prominent reaction was observed in the fatty acids (W1) and amides (W2) region, suggesting that the reaction occurred mainly at level of the cell envelope, which in Gram negative bacteria is formed by the outer membrane, peptidoglycan cell wall and inner membrane.

This surfactant in *L. innocua* induced 100% mortality already at 0.6 mM and at higher concentrations. All stress indexes showed a peak at 0.4 mM and remained on the same values up to 1.0 mM, with the exception of the fatty acids which increased fourfold from 0.8 to 1.0 mM (Fig. 2b). The fact that amides and fatty acids SIs showed higher values than other regions, further fortifies the concept that the cell envelope, containing both types of molecules, was the primary target of this surfactant. In general, *23S-*sh induced the same level of metabolomic reaction in both species, with a size ranging from 0.0 to 6.0, differently from *23S* that was twice more effective in *L. innocua*.

*23S*-12 was the least effective compound in *E. coli* causing a maximum of 25% cell mortality at 1 mM. Carbohydrates (W4), mixed region (W3) and fatty acids (W1) SIs curves showed a peak at 0.8 mM and slightly lower at 1.0 mM. As a confirm that GSI >1 indicates effective cell stress, this index showed values over 1.0 only at 0.8 and 1.0 mM, with 21% mortality (Fig. 1c).

In *L. innocua 23S*-12 killed all cells already at 0.4 mM, proving to be less effective than *23S* and as active as the other two variants (Fig. 2c). Amides (W2), mixed region (W3) and fatty acid (W1) SIs increased following the mortality curve trend while the carbohydrates (W4) SI followed consistently that of GSI.

*23S*-16 showed the same biocidal activity of *23S-*sh in *E. coli*, inducing low mortality rates ranging from 0 at 0.2 mM to less than 75% at 1 mM. All indexes followed the mortality curve trend with the exception of the carbohydrates region (W4), with a maximum peak at 0.8 mM followed by a steep decrease at 1 mM. GSI index showed values over 1.0 only at 0.8 and 1.0 mM, corresponding to 60–75% mortality (Fig. 1d). This compound in *L. innocua* induced 100% mortality at 0.6 mM although, at 0.4 mM, this value was already 80%. GSI showed values equal to 1.0 from 0.4 to 0.8 mM and >1 at 1.0 mM, corresponding to a mortality range from 70 to 100%. The amides (W2) SIs was the most sensitive index for the stress induced by this surfactant, showing the highest values with a size ranging from 0.0 to 6.0 with a general trend similar to that of GSI. The other curves were rather out of phase in comparison with GSI and W2, in fact fatty acids SIs increased from 0.0 to 0.6 mM, decreased at 0.8 mM and then rapidly increased again from 0.8 to 1 mM while carbohydrates were rather low a minimum at 0.4 mM and a maximum at 0.6 mM (Fig. 2d). These data suggest that this surfactant attacks Gram positive and Gram negative cells with rather different mechanism and efficacy, although the cell envelope was one of the targets, as proved by the high response of polysaccharides around 1.0 mM (Figs. 1d and 2d).

### Response Spectra analysis

**Figure 3:**
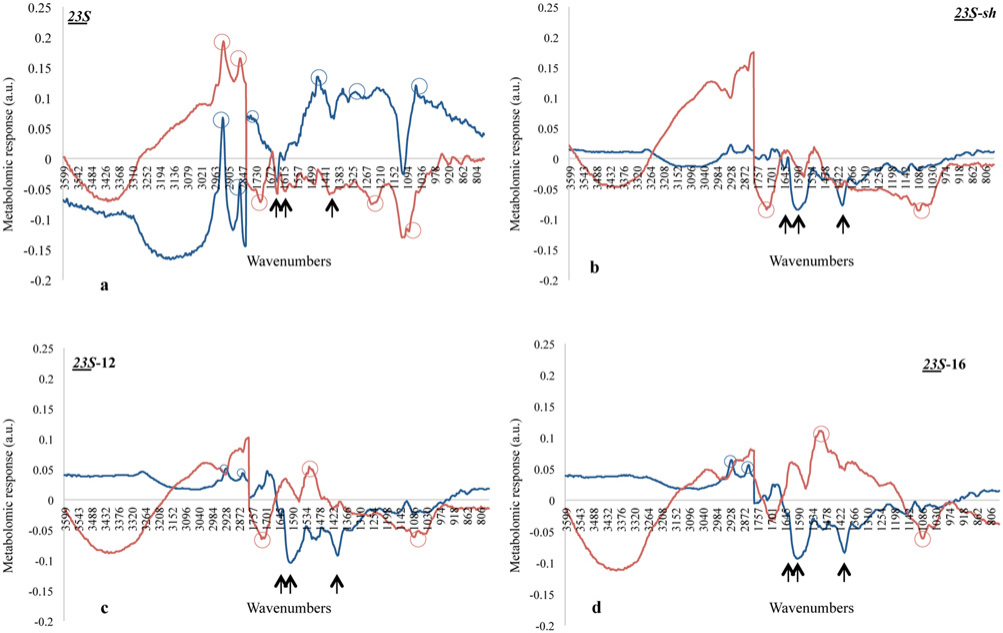
Comparison between the Response Spectra of *E. coli* and *L. innocua* at the surfactants concentrations that determine, respectively, 100% mortality. a.u. stands for *“arbitrary units”*; Panel **a**) RS of *23S* at 0.4 mM for *L. innocua* and 0.6 mM for *E. coli*; Panel **b**) RS of *23S-sh* at 0.4 mM for *L. innocua* and 1 mM for *E. coli*; Panel **c**) RS of *23S*-12 at 0.4 mM for *L. innocua* and 1 mM for *E. coli*; Panel **d**) RS of *23S*-16 at 0.4 mM for *L. innocua* and 1 mM for *E. coli*; Arrows correspond to the wavenumbers referred to peptidoglycan while circles to the wavenumbers referred to the major response peaks in each species. Blue, *L. innocua*; Red, *E. coli*.

The metabolomic effects caused by these surfactants on bacterial cells was more closely studied by displaying the Response Spectra (RS), obtained as difference between the normalized spectrum of cells subject to the tested compound and that of the control maintained in water in the same experimental conditions. This analysis considered only the RSs at concentration causing in each species 100% mortality (see legend of Fig. 3). The degree of variability between RS replicas throughout the FTIR spectra ranged around 3∙10^-3^ for *E. coli* and 3∙10^-4^ for *L. innocua*.

The RSs obtained with the four tested compounds confirmed that *23S* was the most efficient in producing a metabolomic response, whereas *23S-*sh produced a comparatively high response only in the fatty acids region. The general trend of these RSs was compared by Spearman correlation, as a mean to determine the trend similarity independently of the response intensity.

According to this analysis RSs of *E. coli* with *23S* as a similar trend to that generated by *23S-*sh (correlation = 0.875); similarly the correlation of the RSs generated by this species with *23S*-12 and *23S*-16 was 0.811. *L. innocua* produced two similar RSs with *23S-*sh and *23S*-12 (0.882). Interestingly *23S*-12 and *23S*-16 yielded to almost opposite RSs (-0.819) with *E. coli* and *L. innocua*. Weaker correlations were observed in *E. coli* with *23S*-12 against *23S* and *23S-*sh (0.711 and 0.757 respectively); and in *L. innocua* challenged by *23S*-12 and *23S*-16 (0.737). A negative correlation (-0.768) was observed between the RSs obtained by exposing the two species to *23S*-16.

According to the SI analysis presented above, the response to these compounds were concentrated in the W1 fatty acids and in the W2 amides; more rarely some peaks were detected in the W3 and W4 representing mixed region and carbohydrates, respectively. The RSs presented significant reactions of the cells in the W1 region when the cells were exposed to *23S* at 0.6 mM and 0.4 mM (and higher concentrations), respectively in *E. coli* and *L. innocua* (Fig. 3a) and when the latter was challenged by *23S*-12 and *23S*-16 at concentrations from 0.6 mM to 1.0 mM (Fig. 3c and Fig. 3d).

These responses were detected at 2920 cm^-1^ and 2852 cm^-1^ representing the asymmetric and symmetric stretching (ν(CH_2_)) of the fatty acids chains [47]. Interestingly, these two bands were found to be associated to 1741 cm^-1^ (ν(C = O) in lipid esters [47]), as an indication of lipids presents increase in human stem cells [48]. This additional band has been detected in our experiments only when *L. innocua* was exposed to *23S*. This data suggested that the presence of these surfactants caused an increase in the lipid content, presumably those of the external and cell membrane. Another constituent of the cell envelope is the cell wall formed by peptidoglycan (PG). Peaks referring to PG in the RSs were detected at 1657 ~ 1600 and ~1400 cm^-1^ in W2 and W3 regions [49]. Negative peaks at these wavenumbers were detected in both species challenged with *23S* and with *23S*-sh. When *23S*-12 was employed, *L. innocua* showed all the three negative peaks although the first one was weak; *E. coli* instead showed only those at ~ 1600 and ~1400 cm^-1^ (Fig. 3c). Finally, with *23S*-16 *L. innocua* had negative peaks at 1600 and ~1400 cm^-1^ while *E. coli* only at ~1400 cm^-1^. Taken together, these data suggested that surfactants affect negatively the presence of PG in the cell envelope with particular intensity when *23S *was used and less effectively with the other three compounds. These data correlated well with the observation presented above indicating that *23S* was the most effective of the four compounds, being able to kill 80% of *E. coli* and 100% of *L. innocua* at 0.2 mM, whereas at this concentration the other compounds caused much less mortality. Minor variations in the RSs were detected for *E. coli* in the W2 region. Specifically, as a negative peak at 1708 cm^-1^ (ν (C = O) H bonded) when cells were exposed to *23S* and *23S*-sh and as a positive peak at 1515 cm^-1^, representing the shoulder of proteins [47], when cells were challenged by *23S*-12 and *23S*-16. Other potential responses were detected for both species in the mixed region (W3), with *23S *exposure. Namely, in *E. coli* there was a peak around 1240 cm^-1^ (ν (P = O) asymmetric in phospholipids) while in *L. innocua* at ~ 1460 cm^-1^ (δ(CH_2_) of lipids and proteins) and at ~ 1310 cm^-1^ (Amide III) [47]. Finally, the W4 region of *E coli* presented a negative peak at 1085 cm^-1^ (symmetric *ν*(P = O) in nucleic acids and phospholipids [47]) in all tests. *L. innocua* showed a positive response around 1050 cm^-1^ (stretching of phosphate ester [50], only when cells were exposed to *23S*.

### Physical-chemical behavior of *N*-tetradecyltropinium bromide in presence of bacterial cells

Conductometric studies were carried out with the highly biocidal *23S* to verify whether its physical-chemical properties were changed during the interaction with bacterial cells.

**Figure 4:**
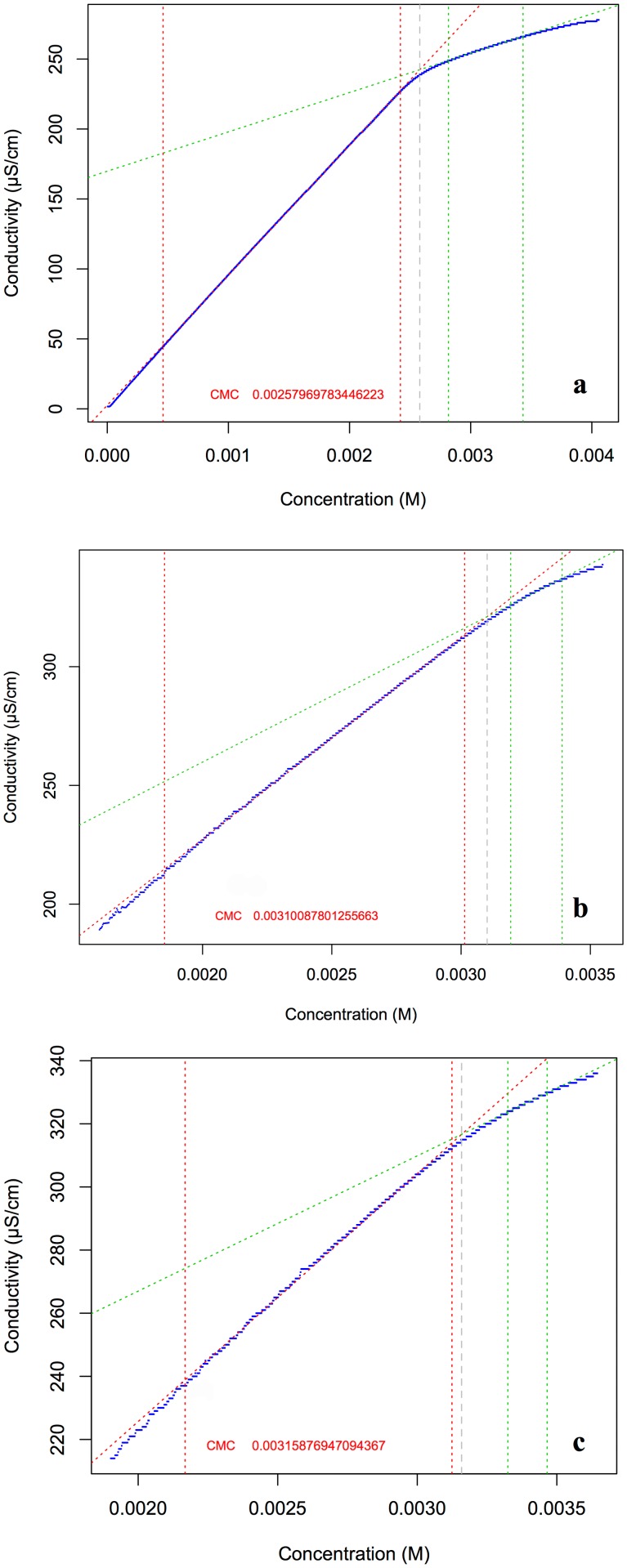
Conductivity profiles of *N*-tetradecyltropinium bromide solution alone and with bacterial cells. Conductivity profiles of *N*-tetradecyltropinium bromide in bidistilled water (a) and in association with *E. coli* (b) and *L. innocua* cell suspension at OD_600_ = 5.0, T = 298.1 K (± 0.1 K). The *c.m.c*. values calculated with CMC-R script were, respectively, 2.58·10^-3^ M, 3.10·10^-3^ M and 3.16·10^-3^ M. *N*-tetradecyltropinium bromide variation coefficient resulted 0.00576; *E. coli* suspension variation coefficient resulted 0.00496; *L. innocua* suspension variation coefficient resulted 0.00511.

The *c.m.c.* of the *N*-tetradecyltropinium bromide solution alone was 2.58·10^-3^ M, whereas in combination with *E. coli* and *L. innocua* suspensions resulted 3.10·10^-3^ M and 3.16·10^-3^ M, respectively (Fig. 4). The presence of bacterial cells shifted the *c.m.c.* by ca 0.5·10^-3^ M, with both species, indicating that the presence of bacterial cells did not change significantly the micellar formation. The little upshift of *c.m.c.* could be justified with the sequestration of the surfactant by the cells.

## Discussion

The efficacy of the tested surfactants changed according to their tail length and to head size, with a maximum devitalizing effect showed by the surfactant with 14C long tail and the tropinium head (*23S*). The reduction of head size in the quinuclidinium surfactant (*23S*-sh) produced a reduction of the antibacterial activity with both species, but particularly with the Gram negative *E. coli* even in comparison with *23S-*12, which has the same tail length. Gram negative bacteria have an external membrane, protecting the peptidoglycan cell wall and the inner membrane, likely to limit the surfactant activity in *E. coli* more than in the Gram positive *L. innocua*, which does not present this periplasmatic space and outer membrane. Interestingly, the *23S*-sh induced the same reduction (-32%) of both biocidal effect and metabolomics stress response (SI) in *E. coli* and a generalized decrease of the metabolomics response in both bacteria. Changes in tail length, by increasing or decreasing it with two methylene groups, caused a reduction of the metabolomics response in both species, especially at low concentrations. The biocidal activity exhibited by *23S*-12 and *23S*-16 was particularly low in *E. coli*, whereas in *L. innocua* 100% mortality was obtained over 0.4 mM. These observations indicate that the metabolomic response was specific of the interaction between the compound and the bacterial species, as postulated in the introduction [8]. In fact, even small structural variation caused quite different effects, both as response extent and as spectral perturbation. Results showed that fatty acids and amides were the compounds more intensively reacting to the surfactant. Another interesting cell component was peptidoglycan, the spectral regions of which reacted more actively in *L. innocua* than in *E.coli.* Peptidoglycan is the main component of the bacterial cell wall and forms a thick multilayer in Gram positive, whereas is a monolayer in Gram negative bacteria. This means that a stronger signal can be expected from *L.innocua* peptidoglycan degradation than from *E. coli*. Moreover, the presence of an external membrane protecting the cell wall in *E. coli* can further explain the lower FTIR signal attributed to the peptidoglycan degradation.

Using these compounds, we were able to observe three types of metabolomic response. The first and most common type of response was observed in the majority of the experiments when the metabolomics response progressed along with cell mortality, indicating that cells tried to counter the surfactant actively, but were overwhelmed by it. In a second type (*23S*-sh against *E. coli*) the response was particularly strong at low surfactant concentration that induced less than 2% mortality, indicating that cells tried somehow to contrast the devitalization, although at higher concentration the trend of cell killing and metabolic responses were almost parallel. A third response type was observed when cell mortality resulted higher than the metabolic response, i.e. with *L. innocua* challenged by *23S*-sh and *23S*-16. In the former case the cell mortality was 100% at 0.2 mM with a weak metabolomics response (GSI = 0.650); in the latter the total culture devitalization could be obtained at 0.6 mM with GSI = 1.008. This third type of behavior is likely to indicate the cases in which the surfactant has a strong and immediate action without an effective response of the metabolome.

The above observations on these three different modes of action were further corroborated by correlating the whole spectrum response and the cell mortality at the lowest concentrations inducing 100%, or the highest observed level of mortality. The first type of behavior showed figures ranging from 0.88 and 0.93 in *E coli* with *23S*, *23S*-12 and *23S*-16. Similarly, 0.78 and 0.82 were obtained with *L. innocua* challenged by *23S*-sh and *23S*-12. The second type of response (*E. coli* with *23S*-sh) yielded 0.429 correlation, whereas the third behavior (*L. innocua* with *23S* and *23S*-16) was characterized by 0.65 and 0.69 correlation values respectively. Although this aspect requires further systematic investigations, it seems that the first type of response (mortality similar to cell response) can be individuated by correlation values no lower than 0.7, whereas the second (more response than mortality) and the third (more mortality than response) are characterized by correlations respectively below 0.5 and between 0.5 and 0.7. These obviously tentative and indicative thresholds probably split a *continuum* set of behaviors, as witnessed by the fact that *L. innocua* with *23S*-16 had a borderline correlation (0.69) and in fact the cell mortality grew more rapidly than the metabolomic response, but the GSI reached the 1.008 value. However, a deeper insight in this parameterization can lead to helpful tool to individuate in a quantitative way the relation between mortality and metabolomics response induced by cell-stressing agents. These parameters should be optimize to characterize not only new surfactants, and their variants, but any type of biocidal molecule, in order to maximize the devitalization and reduce as much as possible the “*ante mortem*” response, which indicates the effect of cell resistance prior to death.

When the surfactants induced the mortality at low concentration (e.g. *23S* with both species) it was possible to observe an increase of the metabolomics response at higher concentration, which is not likely due to the metabolic activity, being the cells dead. This suggests that part of the metabolomic change is due to the *ante mortem* cell response and part to *post mortem* chemical reactions occurring in the dead cells caused by the direct interaction of the surfactant with the cell materials. Alternatively the mix of various cell components could induce these changes once the surfactant has caused the cell components to mix up. Surfactants in environmental sanitization should couple the high biocidal effect with to low *ante mortem* and high *post mortem* metabolomics change, being the latter an evidence of cell disruption, prodromal to the whole cell removal.

The conductometric analyses have shown that the presence of cells did not change the *c.m.c.* significantly, indicating the physical-chemical properties of the surfactant remained unchanged. The little *c.m.c.* increase observed could be due to the absorption of surfactant monomers by the cells, thus shifting the formation of micelles. Moreover, the *c.m.c.* increase can be partly due to the release of salts from the cells to the solution caused by the membrane permeabilization. These findings indicated that the biocidal activity was exerted at concentrations well below the *c.m.c.* and should therefore be ascribed to the surfactants monomers.

Some of the variants tested in this study gave different results against the two bacterial species. At the same time, the *23S* was the most efficient with both bacteria and yeasts [40]. These findings indicate that surfactant structure influences both the efficacy and the spectrum of action, suggesting that compounds tailored for the various situations can be produced once a better understanding will be gained on the relation between structure and biological activity taking in consideration the huge potential of synthetic chemistry combined with microbial biodiversity.

## Supporting Information

S1 Table Structures of the amphiphiles. Chemical structures of the surfactants used in this work, names and acronyms.
(DOCX)
