# Supplementary material for: FTIR Metabolomic Fingerprint Reveals Different Modes of Action Exerted by Structural Variants of N-Alkyltropinium Bromide Surfactants on Escherichia coli and Listeria innocua Cells
Source: PLoS One. 2015 Jan 14;10(1):e0115275. doi: 10.1371/journal.pone.0115275 (PMC4294686; doi:10.1371/journal.pone.0115275)
Supplement: S1 Table — Chemical structures of the surfactants used in this work, names and acronyms. (DOCX) [file pone.0115275.s001.docx]

**Table S1.** Structures of quaternary ammonium salts.

|  |  |
| --- | --- |
| ***23S*-12** |   *N*-dodecyltropinium bromide |
| ***23S*** |   *N*-tetradecyltropinium bromide |
| ***23S*-16** |   *N*-hexadecyltropinium bromide |
| ***23S*-sh** |   *N*-dodecylquinuclidinium bromide |
